# Novel Assessment Methodology for Laser Metal Deposition of New Metallic Alloys

**DOI:** 10.3390/ma16020636

**Published:** 2023-01-09

**Authors:** Xabier Cearsolo, Mario Arrue, Maitane Gabilondo, Jon Mikel Sanchez, Haize Galarraga, Maider Garcia de Cortazar, Franck Girot Mata

**Affiliations:** 1 Department of Additive Manufacturing, IMH Campus, Azkue Auzoa, 1, 20870 Elgoibar, Spain; 2 TECNALIA, Basque Research and Technology Alliance (BRTA), Paseo Mikeletegi, 2, 20009 Donostia, Spain; 3 Department of Mechanical Engineering, Faculty of Engineering of Bilbao, University of the Basque Country (UPV/EHU), Plaza Ingeniero Torres Quevedo, 1, 48013 Bilbao, Spain; 4 Ikerbasque, Basque Foundation for Science, Euskadi Plaza, 5, 48009 Bilbao, Spain

**Keywords:** additive manufacturing, metal powder, assessment methodology, laser metal deposition

## Abstract

Metal additive manufacturing technologies are gaining great interest. However, the existing metallic alloys are generally formulated for conventional manufacturing processes. Thus, it is necessary to adapt their chemical composition or develop new alloys for the manufacturing conditions of additive manufacturing processes. The main method for manufacturing metal powder is gas atomization, but it is very expensive with long manufacturing times. Therefore, it is necessary to develop alloy validation methods that simplify the development process of new alloys. This paper deals with a methodology based on thermodynamic heat transfer equations, simulation, and powderless tests. This novel methodology enabled the determination of the optimal conditions for the laser melting deposition process of the commercial AA7075 alloy with a reduced number of experimental tests with powder, reducing the difficulties inherent to powder processing. The developed process was divided into two stages. In the first stage, the heating of the substrate was studied. In the second stage, the depositions of single tracks were validated with the parameters extrapolated from the previous stage. Hence, it was possible to manufacture single tracks free of cracks with an adequate aspect ratio.

## 1. Introduction

Over the last decade, Additive Manufacturing (AM) technologies have gained increasing attention due to their capability to manufacture complex, lightweight, and economical parts with short lead times [1,2,3,4,5]. In contrast to subtractive methods, AM processes consist of the manufacturing of 3D parts layer by layer. According to ASTM F2792-12 standards [6], AM is defined as a process of joining materials to make objects from 3D model data, usually layer upon layer, as opposed to subtractive manufacturing methodologies. The different AM techniques can be classified into seven categories: VAT Photopolymerization, Material Jetting, Binder Jetting, Material Extrusion, Powder Bed Fusion (PBF), Direct Energy Deposition (DED), and Sheet Lamination. PBF and DED are the most common processes for the additive manufacturing of aluminum alloys. These technologies are gaining relevance in many industrial sectors, such as automotive, aeronautic, and medical because they enable the manufacturing of complex parts that cannot be fabricated with traditional technologies [1,7,8,9,10].

The variety of materials that are processed by AM is very wide, among which are plastics, metals, and ceramics. Metallic alloys are widely used for their superior mechanical properties when they are manufactured by different AM techniques [11]. However, most of the metallic alloys used nowadays were developed for conventional manufacturing processes, with different melting and solidification properties from the AM technologies [3]. Laser-based metallic AM processes are characterized by complex thermal histories that involve directional heat extraction, high thermal gradients, and rapid solidification, with cooling rates on the order of 10^5^–10^6^ K/s [12]. In addition, the repeated melting and tempering cycles of the layer-by-layer AM manufacturing produce microstructures and properties different from those obtained in conventional processes [13]. As the use of traditional aluminum alloys in AM may lead to parts with inappropriate microstructures and cracks [3,14,15], new alloys must be developed for the specific manufacturing conditions of AM processes. This requires an alloying strategy to adequate the composition of conventional alloys to the processing conditions of AM and/or the development of novel alloys that are more appropriate for these processes [15]. 

The process of designing new alloys for some AM processes usually starts with the determination of the composition, which is followed by the transformation of the alloys from casting ingots to AM raw materials format. Powders are the most used raw material for AM technologies. Currently, the manufacturing of powders with new compositions is a key factor hindering the growth of AM [16]. The major manufacturing route of AM powders is atomization since powders synthetized by this technology have a spherical morphology, which is preferred because of its favorable flow and uniform packing properties. However, the conventional approach of manufacturing a set of compositions and testing them becomes cost prohibitive, as the powder atomization processes have very high prices for low material quantities [14,17,18]. Consequently, new validation or assessment methodologies must be developed to be able to make a preliminary selection before final validation via additive manufacturing processing. Ewald et al. [19] demonstrated that one approach for the development of new alloys is the dry mixing of powders by manufacturing powder blends. The use of blends avoided the step of powder atomization, which is usually time-consuming and expensive. Chen et al. [20] presented a review on the development of metallic alloys using elemental powder blends by Laser Metal Deposition (LMD). The critical technical challenges, mainly in composition control, were also discussed for future development. Stopyra et al. [21] demonstrated a powderless approach to determine the parameter window for the processing of alloys by Laser Powder Bed Fusion (L-PBF). They carried out single-track melting of an AA7075 substrate to establish the range of energy density that enables obtaining a stable melt pool. Zhao et al. [22] presented Melt Spinning (MS) as an alternative process to study the microstructures and phases of parts manufactured by selective L-PBF because the cooling rates of both processes are very similar and the raw materials required for the melt spinning process are not so demanding. The sample fabricated by MS showed a similar supersaturated structure to the PBF counterpart and both samples exhibited analogous microstructures. Sinclair et al. [23] used water-atomized powder as raw material and developed a feeding technology for Powder Bed Fusion (PBF) that used vibration to control the flow of powders. They used low ‘spreadability’ water-atomized metal powders owing to their significantly lower cost in comparison to gas-atomized powders.

Simulation tools can also be used to simplify the procedure for process parameters’ determination in AM. Soffel et al. [24] performed the optimization of remelting process parameters for part repair in DED via numerical simulation. The models predicted the melt pool shape, and they concluded that the optimized remelting parameters increased the bonding quality between the base and deposited materials. Ponomareva et al. [25] studied the Wire Arc Additive Manufacturing of an Al-Mg alloy, where the thermal state was estimated by finite element simulation, which was further verified by comparison with thermocouples data.

To tackle the issues above, and because of the great potential of AM processes, a validation method to simplify the development process of new alloys for AM, specifically LMD, was developed. The novel assessment methodology was validated with the AA7075 aluminum alloy because it is extensively used in aircraft structural parts in highly stressed structural applications. However, this alloy presented some challenges for the processability of LMD related to its susceptibility to hot cracking. AA7075 alloy is regarded as one of the strongest commercial aluminum alloys due to the high content of Zn, Cu, and Mg. The high strength is mainly attributed to the precipitation of the MgZn_2_ (η-phase), Al_2_Mg_3_Zn_3_ (T-phase), and Al_2_CuMg (S-phase) intermetallic phases after the subsequent heat treatment. On the other hand, the chemical composition of this alloy leads to a large solidification range which improves the susceptibility to hot cracking of the alloy. Therefore, recently, efforts were made to improve the processability of AA7075 alloy by laser, which were based on the modification of the chemical composition. Khalil et al. [26] studied the influence of adding different alloying elements on the microstructure and mechanical properties of AA7075 material to improve the laser weldability in AM. They carried out the laser melting on as-cast AA7075 alloy with four different alloying elements. The results showed that the grain size decreased significantly after the AA7075 alloy was modified by the addition of rare earth elements. However, the microstructural analysis revealed the presence of solidification cracks. Mosleh et al. [27] also investigated the effect of adding modifying elements to as-cast AA7075. They conducted the melting of three different casted alloys that included, in all cases, 4% Si. The results showed that Si had a significant impact on the absence of hot cracking during solidification in laser melting. Alternatively, the present work shows a novel methodology that can process the AA7075 alloy by laser without the need to change the chemical composition to avoid hot cracking. The methodology presented herein allows the validation of metallic alloys in a simplified way by avoiding the use of powders in the first stages. This reduces the number of alloys manufactured in the form of powder and the quantity of them, reducing development time and costs. The selection of the initial conditions was conducted by integrating thermodynamic heat transfer equations and computational tools, which facilitated the process of determining the parameters window. Finally, the optimized parameters were used for the LMD process of the alloy. 

## 2. Materials and Methods

**Figure 1:**
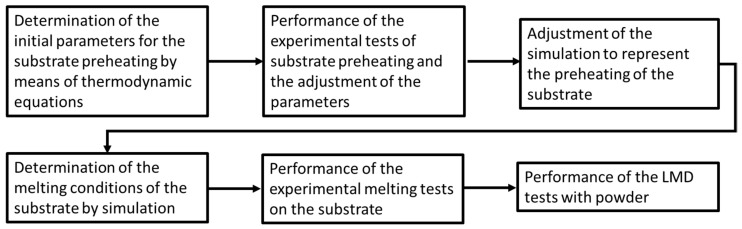
Summary of the developed methodology.

In the first stage, substrate samples were melted with a laser beam in an Additola LMD cladding machine. The heat input process was divided into two steps, preheating and melting, to reduce the thermal jump to melt the material and improve the absorptivity. Initially, preheating experiments were carried out based on the parameters determined by thermodynamic heat transfer equations. After the selection of the preheating parameters, the simulation study was performed by Wincast expert (RWP, Roetgen, Germany) simulation software. The simulation parameters were adjusted to the experimental results to represent the thermal processes of the substrates by simulation. Then, the simulation tool was used to determine the initial parameters of the melting stage. After selecting the most suitable parameters for the preheating and melting of the substrate, LMD tests were carried out to study the feasibility of the proposed methodology. In Figure 1, the summary of the developed methodology is summarized. 

### 2.1. Substrate Heating

The raw material consisted of wrought alloy sheets. It was necessary to machine the sheets to obtain suitable substrates for the experiments. The sample should not have a large cross-section to concentrate the energy and avoid heat losses through the sample. This is especially important for aluminum alloys due to the high thermal conductivity of the material [28,29]. However, the substrate should have enough height and width to differentiate the molten area from the unaffected zone. Furthermore, one of the directions, which would correspond to the direction of laser scanning, must be kept long enough so that a characterizable melt zone could be generated. Based on these criteria, the selected geometry was 10 × 10 × 50 mm^3^. 

The experimental procedure of substrate heating was divided into two steps: preheating and melting. The first was conducted to reduce the thermal jump to melt the material, improve the absorptivity so the energy necessary for the melting step was reduced, and reduce the residual stresses during melting [28]. Absorptivity of aluminum increases with temperature [30,31].

#### 2.1.1. Substrate Experimental Preheating

The temperature increase in the preheating step was set at 300 °C, so that the temperature of the substrate was closer to the solidus temperature of the alloy (T_solidus_ = 475 °C [21]) without exceeding it. Not reaching the solidus temperature values avoided possible microstructural changes during preheating. 

An approximation of the heat that must be supplied to the substrate to produce an increase of 300 °C was calculated with a thermodynamic heat transfer equation (Equation (1)):

(1) $$Q=\rho \cdot c_{p}\cdot V\cdot \Delta T$$

where ρ is the density of AA7075, *c_p_* is the thermal capacity (ρ·*c_p_* is 2.63 J/cm^3^·K), *V* is the volume of the substrate (5 cm^3^), and Δ*T* is the temperature rise (300 °C). The approximation of the heat that must be provided to the substrate to reach the set temperature increase is shown in Equation (1). Since this energy must be provided by the laser, the time required for preheating the sample was calculated by Equation (2):

(2) Q=α·P·t

where *P* is the LMD equipment power (W) with a maximum power of 1000 W, *t* is time (s), and α is the absorptivity coefficient of the alloy (assumed value of 16%). Thus, the time required to supply 3950 J with the laser was 24.7 s, as shown in Table 1. 

Regarding the diameter of the beam, to boost a more uniform heat distribution on the surface of the probe, a laser beam diameter of 10 mm was established, coinciding with the width of the substrate. The focal distance was calculated using the Rayleigh length (8.3 mm) of the equipment, which was 146 mm in this case. Furthermore, the working area was restricted to a central area of 40 mm long, so that the remaining 5 mm from each end did not correspond to the area to be treated. To reduce the temperature difference between the start and end points of the sample during the preheating, the preheating was carried out in several scans to obtain a more uniform temperature across the substrate. Initially, six scans were performed (three roundtrip). As the total preheating time had to be 24.7 s and the total length was 240 mm for six scans (6 × 40 mm), the corresponding speed was 580 mm/min. The selected conditions for the initial preheating experiments are summarized in Table 2.

**Figure 2:**
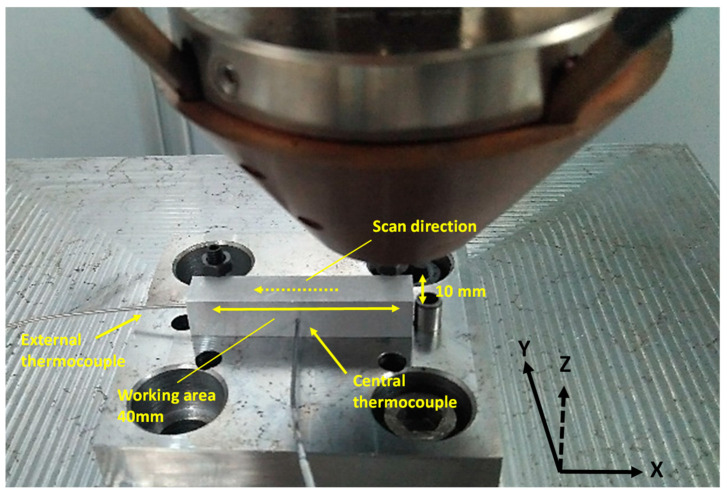
Experimental set-up of the positioning of the samples with the thermocouples on the LMD base.

As mentioned above, the purpose of preheating was to achieve a temperature increase of 300 °C. Therefore, it was necessary to measure the temperature during the test to verify that the desired increase was achieved. There are different ways to measure temperature including thermal imaging camera or thermocouples [32,33,34]. On one hand, it was proposed to use a thermal imaging camera, but the high reflectivity of aluminum represented a problem to make the measurement. Therefore, it was decided to measure the temperature using K-type thermocouples. To overcome the challenge of attaching the thermocouples on the surface of the samples, two holes of 1.2 mm in diameter and 5 mm in depth were machined in the centre and at the end of the substrate to insert the thermocouples. Figure 2 shows the location of the holes. One of the holes was in the middle of the X–Z plane and the other was in the Y–Z plane. Temperature changes were monitored and recorded by a DATAQ Instruments Data Logger at a sampling frequency of 0.02 s. After machining the parts with the holes and cleaning the surface with isopropanol, the samples including the thermocouples were placed on the base, as illustrated in Figure 2. Then, the preheating test with six scans was performed with the previously defined parameters in Table 2. 

The temperatures reached with the conditions of Table 2 were excessively high, especially in the center of the sample, where the T_solidus_ of the AA7075 alloy was exceeded. Therefore, it was decided to reduce the number of scans, and with that, the preheating time. As shown in Section 3.1.1, a temperature increase of 300 °C was achieved without reaching the T_solidus_ for a total of four scans, which was selected for future experiments. Table 3 shows the newly selected conditions. To ensure the repeatability of the results, three trials were carried out under the same conditions. 

#### 2.1.2. Substrate Preheating Simulation

To facilitate the selection of the conditions of the experiments in the melting stage, the WinCast expert simulation software was used. The simulation was performed with the same geometry as in the experimental tests, which was placed on a steel table considering conduction heat transport from the sample to the table and convection from the sample to the air. The heat transfer values were determined according to Wincast software, which corresponded to Al-steel contact and an air speed of 0.1 m/s. The spot size was 0.6 mm with a penetration in the material of 0.6 mm and with the same speed as in the experiments. The mesh size was established at 0.2 mm with tetrahedral geometry. The material properties were obtained from the commercial Wincast software database. 

First, the simulation conditions were adjusted to represent the temperatures obtained during the preheating step. The initial conditions of the preheating with four scans were simulated considering an effective power of 160 W (absorptivity of 16%), i.e., the same as in Equation (2). However, the temperatures reached with the simulation were lower than the temperatures measured experimentally. Consequently, the simulation power was adjusted to 400 W (absorptivity α of 40%) so the power ratio of P_experimental_ and P_simulated_ was approximately 2.5 (Table 4). 

#### 2.1.3. Substrate Melting

Once the preheating conditions were determined and the simulation parameters were adjusted, WinCast expert was used to determine the experimental conditions for the sample melting. As a criterion for simulation, it was established not to exceed the evaporation temperature of any alloying element of the AA7075 alloy. Among the different components of the alloy, zinc and magnesium have the lowest evaporation temperature [28]. Consequently, it was determined that the maximum temperature should not exceed 907 °C at any point on the sample. Another condition included in the simulation was to obtain a temperature higher than the T_liquidus_ (635 °C) of the alloy from the beginning of the melting path to form a molten pool throughout the entire work area. 

After performing several tests in the simulation, a configuration of parameters that met the established criteria was obtained for the melting step. These parameters corresponded to a simulation power of 400 W, a scan speed of 300 mm/min, and a laser spot of 0.6 mm (Table 5). 

**Figure 3:**
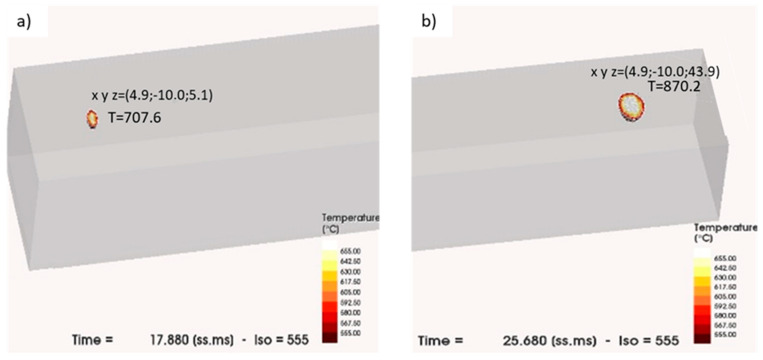
Representation of the simulation at (**a**) the beginning and (**b**) the end of the melting step.

The simulation of the heating of the substrate with the preheating parameters in Table 4 followed by melting parameters in Table 5 is shown in Figure 3. The images represent the melt pool at the beginning and the end of the melting step. According to the previously defined melting conditions to avoid the evaporation of elements, the temperature at the beginning and at the end of the path was 707 °C and 870 °C, respectively.

After obtaining the appropriate simulation conditions for the melting step, the previously determined ratio of P_experimental_ and P_simulated_ was used to calculate the power to be used in the experimental tests. Since the power in the simulations was 400 W, a power of 1000 W should be used in the experiments. Based on the power and scan speed of the simulation, the energy per unit length (*E_L_*) parameter was determined by Equation (3) [35]:

(3) $$E_{L}=\frac{P}{v}$$

Applying this equation to the experimental conditions, an *E_L_* value of 200 J/mm was obtained. Taking this value as a reference, experiments were performed to study the effect of power and scan speed on the melt pool, keeping the *E_L_* constant. The conditions of the experiments window are shown in Table 6. The test with each treatment was repeated three times to analyse the repeatability of the experiments. 

**Figure 4:**
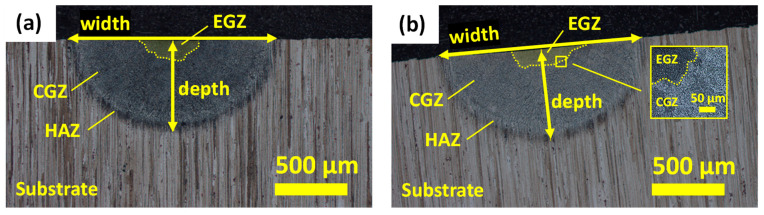
Optical images of the microstructure and measurements of the melt pool for treatment T1 in (**a**) initial and (**b**) final zones of the sample.

After the preheating and melting experiments, the metallographic analysis of the samples was carried out to determine the properties of the molten pool and the thermally affected area. The surface of the processed samples was analysed with an optical microscope. Then, the melt pool was characterized, for which the cross-section of the beginning and the end (10 mm from the edge) was cut, ground, polished, and etched. Finally, the melt pool dimensions were determined by ImageJ (University de Wisconsin—Madison, Madison, USA) software. Figure 4 shows the microstructure of the melt pool for treatment T1 in the initial and final zones of the sample. The melt pool shows three different regions in the cross-section of the analysed sample. The heat-affected zone (HAZ), the columnar grain zone (CGZ), and the equiaxed grain zone (EGZ) regions were differentiated. 

### 2.2. Laser Metal Deposition of AA7075 Powder

The LMD tests were conducted with AA7075 powder (Sichuan Hermus Technology, Chengdu, China) with a particle size of 50–150 µm. The chemical composition of the powder in wt. % provided by the supplier is shown in Table 7. 

#### 2.2.1. Inert Gas Chamber Design

**Figure 5:**
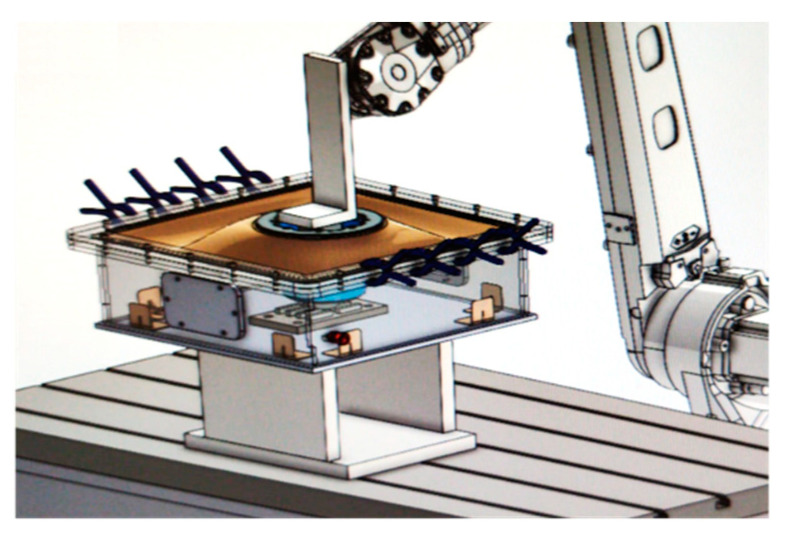
Photograph of the design of the inert chamber.

As aluminum powder can be flammable and explosive in the presence of oxygen and a heat source, an inert chamber had to be used to control the oxygen content in the LMD experiments with powder. After a literature search [28,36,37,38,39], it was decided to fabricate an inert chamber with a rigid box and a flexible lid. Eventually, a methacrylate box with an area of 500 × 500 mm^2^ and a height of 150 mm was made. The lid was made of flexible rubber so that it allowed closure between the head and the edge of the box, and it also enabled the movement of the laser head in all directions. The design of the fabricated inert chamber is shown in Figure 5. 

**Figure 6:**
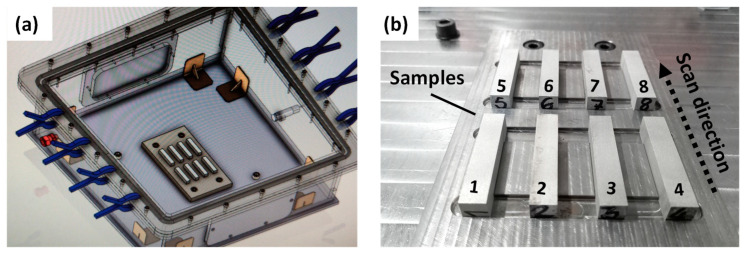
(**a**) Experimental set-up of the methodology and (**b**) sample location inside the inert chamber.

A stainless-steel plate was machined to place the samples during the experimentation as shown in Figure 6a. In addition, an oxygen sensor connected to an Arduino Nano was integrated into the box. This was placed at a level slightly higher than the surface of the substrate due to the higher density of argon compared to air. The set point value for the sensor was an oxygen concentration of 0.2%. At higher levels, the Arduino activated a solenoid valve that opened the argon inlet to the box. The sample location inside the inert chamber and the scan direction are shown in Figure 6b.

#### 2.2.2. LMD Experiments 

Experimental tests were defined using the process selected in Section 2.1.3 applying variable powder feed rates and repeating each experiment twice. The process conditions for each experiment for powder deposition of AA7075 are summarized in Table 8. 

Finally, the topology of the deposited single tracks was measured using a 3D optical surface metrology system model DCM 3D (Leica, L’Hospitalet de Llobregat, Spain) in conjunction with the Leica Map DCM 3D software. The cross-section of the deposited single tracks was characterized by optical microscopy. Vickers microhardness FM-700 model (FUTURE-TECH, Kawasaki, Japan) was employed on the polished sample surface using a 0.1 kg load, applied for 10 s. At least 10 random individual measurements were made. 

## 3. Results

### 3.1. AA7075 Substrate Heating

#### 3.1.1. Substrate Preheating

**Figure 7:**
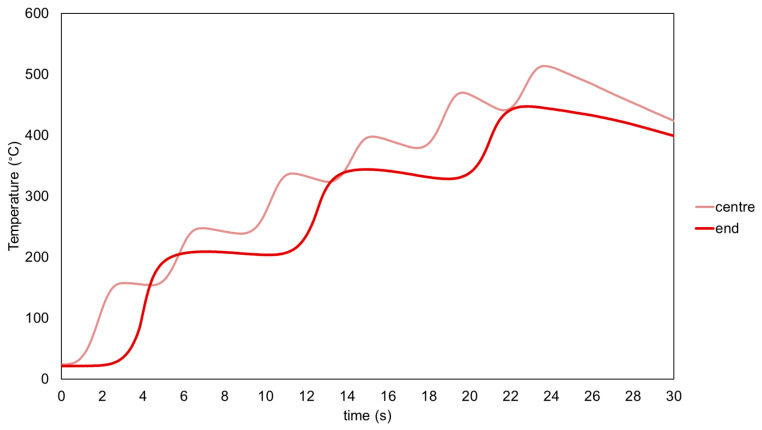
Evolution of temperature as a function of time during the preheating (light red line in the centre, dark red line in the end).

First, the preheating test was carried out on the sample of 10 × 10 × 50 mm^3^ with the parameters summarized in Table 2. The purpose of this preheating was to increase the temperature of the substrate to 300 °C. Therefore, the temperature during the experiment was measured with two thermocouples located in the centre and end of the substrate as is shown in Figure 2. In Figure 7, the evolution of the temperature as a function of time during the preheating experiments in both thermocouples is plotted. According to the diagram (Figure 7), the central thermocouple had six peaks, which corresponded to the six scans (three roundtrip), whereas the thermocouple in the end only presented three peaks. Regarding the temperature values, it was observed that in the central thermocouple, the maximum temperature was higher than at the end. This is because, in the last scan, the laser passed through the centre on the return, increasing the temperature. 

Table 9 shows the maximum temperatures in the position of the thermocouples. In both cases, the target temperature of 300 °C was significantly exceeded. 

**Figure 8:**
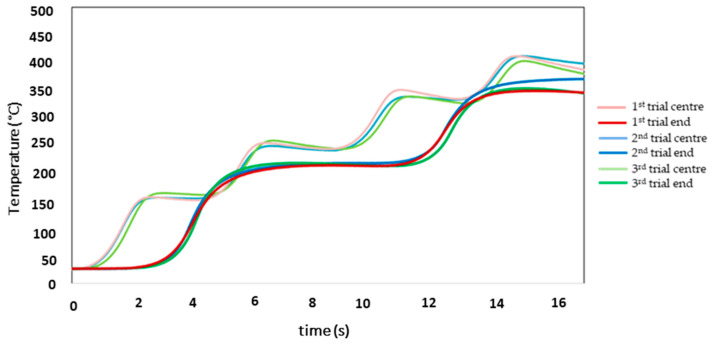
Evolution of temperature as a function of time for the three trials with four scans.

As the temperatures for the six scans exceeded the T_solidus_ of the alloy, the number of scans was reduced to four. The evolution of the temperature as a function of time for the three trials with the parameters of Table 3 is shown in Figure 8. The curves of the temperatures reached in the three experiments were similar—an indication of good repeatability. 

Table 10 shows the average values of the maximum temperatures for the experiments with four scans. As expected, based on the results of the six scans, after four scans the rise of 300 °C was reached, both in the central and external thermocouples. In the case of the central one, although the temperature increase was greater than the established one, it was far from reaching the T_solidus_ of the alloy. As the desired temperature increase (300 °C) was achieved in the experiments with four scans, but without reaching T_solidus_, these preheating conditions were selected. 

#### 3.1.2. Substrate Simulation 

**Figure 9:**
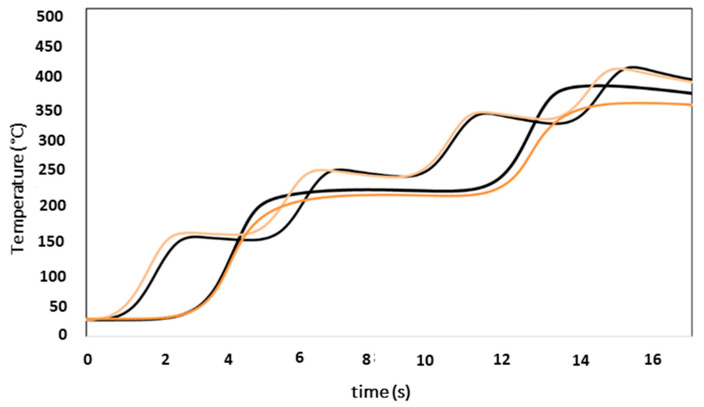
Representation of average experimental temperatures (light orange line corresponds to the centre and the other to the end) and simulated temperatures (black line) with WinCast expert.

In the next stage, the Wincast expert simulation software was used to facilitate the selection of the melting conditions of the experiments. Therefore, the simulation had to be adjusted to the experimental results. The results of the simulations carried out with the parameters in Table 4 were compared with the average experimental temperatures shown in Figure 8. As mentioned in the experimental procedure, the simulation power was adjusted to 400 W. Therefore, the power ratio of P_experimental_ and P_simulated_ was 2.5. The temperature values of the simulation at the centre and at the end of the sample agree with the experimental results (Figure 9). 

#### 3.1.3. Substrate Melting

**Figure 10:**
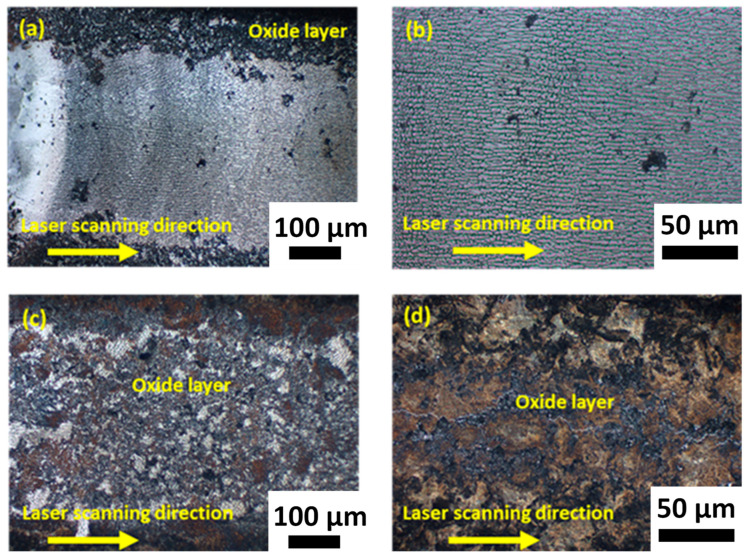
Optical micrographs of the surfaces of the substrates treated with (**a**) and (**b**) T1, (**c**) T2, and (**d**) T3 treatments.

Once the preheating conditions were determined and the simulation parameters were adjusted, WinCast expert was used to determine the experimental conditions for melting. The selected parameters used are shown in Table 6. The samples treated with these parameters were analysed to determine the properties of the molten pool and the thermally affected area. First, the surface appearance of the substrates with different treatments was determined by optical microscopy in Figure 10. The images show that an oxide layer was formed because of the laser treatment and its thickness increased with decreasing power and scan speed. Under the conditions of T1 (Figure 10a,b), the oxide layer formed in the boundaries of the melted zone, whereas for T2 (Figure 10c) and T3 (Figure 10d), the layer completely covered the surface. 

**Figure 11:**
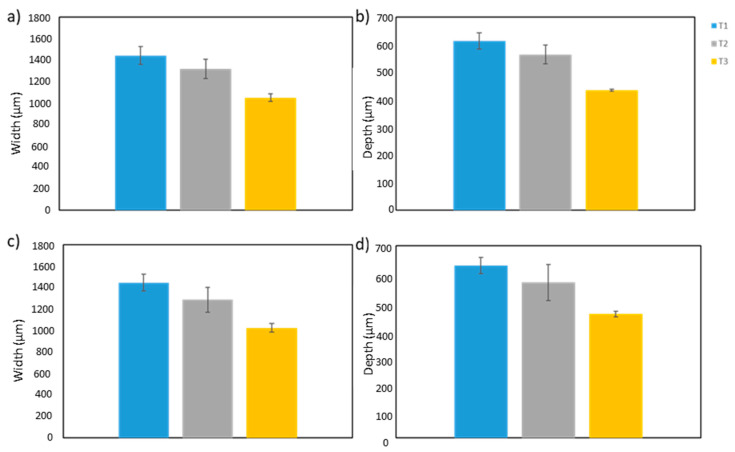
Melt pool (**a**) width at the beginning, (**b**) depth at the beginning, (**c**) width at the end, and (**d**) depth at the end.

After analyzing the surface appearance, the cross-section of the samples was studied. The width and depth of the melt pool were determined at the beginning and the end (10 mm from the edge) of the substrate. According to the results in Figure 11, treatment T1 provided the largest pool sizes, both at the beginning and the end, followed by treatments T2 and T3. Although the total energy input in all cases was the same, the results showed that the scan speed and power values affected the melt pool size. In treatment T1, for the same energy input, the treated area was larger than for the other two treatments. 

It is also worth mentioning that although AA7075 alloy is prone to hot cracking, in this work, the presence of cracks was not observed for any of the conditions studied. Therefore, the methodology developed in the work was adequate to avoid hot cracking in the AA7075 alloy. The melting of AA7075 alloy without hot cracking was previously reported in the literature [27], but the composition of the standard alloy was modified by the addition of different amounts of Si and other alloying elements.

Based on the surface appearance and melt pool size, the selected treatment for the LMD deposition of AA7075 powder was T1. This treatment consisted of preheating, followed by melting using a power of 1000 W and a speed of 300 mm/min. The surface of the substrate treated using these conditions was not covered entirely by an oxide layer, unlike T2 and T3. Furthermore, the treated area was the largest among the studied treatments. 

### 3.2. Laser Metal Deposition of AA7075 Powder

**Figure 12:**
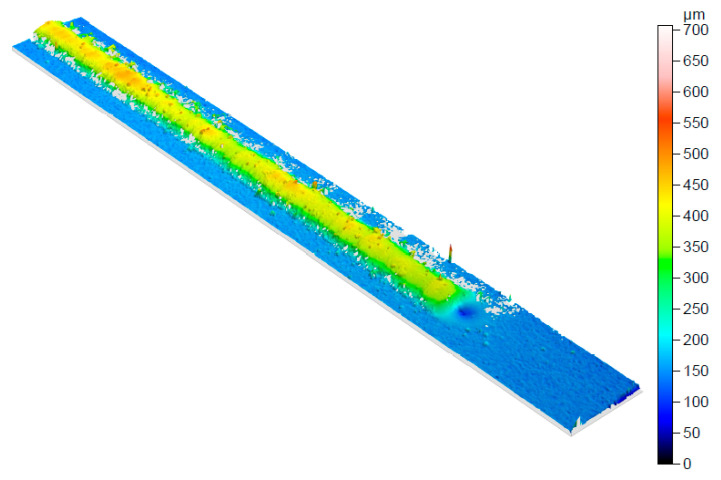
Qualitative 3D appearance of the single-track topology.

The topology of the deposited single tracks was measured using a 3D optical surface metrology system. The qualitative 3D appearance of the beads is shown in Figure 12. The topology shows a greater depth at the starting point of the test. This is due to the volumetric change after the melting of the material cannot be filled by the material in this zone. In general, the topology showed a similar overall height and width in the whole deposited surface. 

In Table 11, the experimental results of height and width determined by quantitative topology measurements were used to calculate the aspect ratio of the single tracks for the studied conditions. The conditions that were in the range of the optimal aspect ratio value of 4–6 [40] were those of a powder flow of 1.4 g/min, 5.8 being the obtained aspect ratio. 

**Figure 13:**
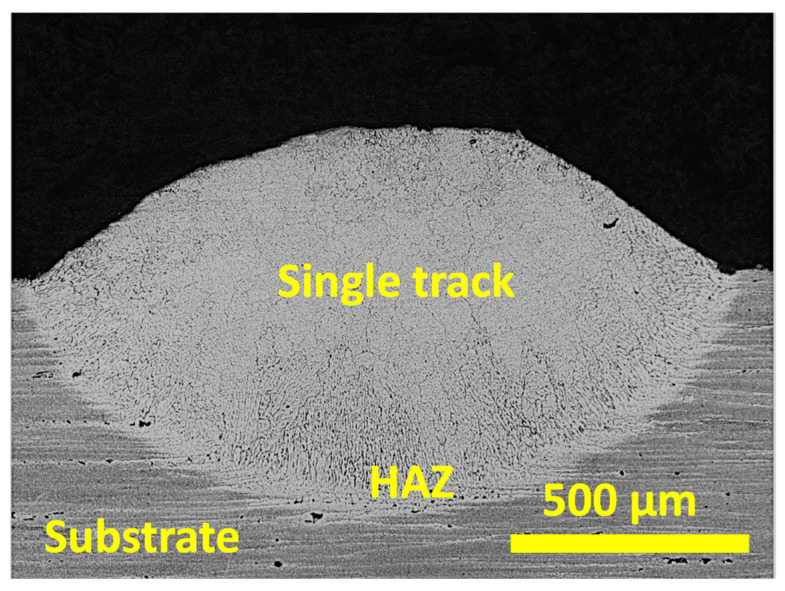
Representative optical micrograph cross-section of the samples manufactured with a powder feed rate of 1.4 g/min.

Figure 13 shows a representative cross-section micrograph of the single track deposited with a feed rate of 1.4 g/min. The experimental conditions resulted in a microstructure free of cracks, avoiding the hot cracking tendency of the AA7075 alloy [41,42,43,44]. Therefore, the methodology presented in this work was adequate to obtain single tracks without cracks and with an adequate aspect ratio.

Finally, the microhardness of the deposited single tracks was evaluated in three different zones of the cross-section of the samples. The mean microhardness values with the standard deviation are shown in Table 12. The experimental microhardness values of the different zones are in accordance with those obtained in the literature [26,45,46]. The difference between the deposited single track and the substrate was attributed to the evaporation of the strengthening elements in AA7075 alloy [26].

## 4. Conclusions

A novel methodology was developed to determine the conditions for the deposition of single tracks by the LMD process of the AA7075 alloy with a reduced number of experiments with powders, reducing powder consumption and other inherent difficulties. 

The parameters determined in the heating stage of the substrate and extrapolated to the LMD tests with powder were suitable for obtaining single-track deposition of materials without defects and with an adequate aspect ratio. In addition, the simulation tool used in the heating stage of the AA7075 substrate was adequate to represent the preheating of the substrate, which allowed it to be used as a tool to select the melting conditions, reducing the number of experimental tests with powders. Further work, with the aim of solving other key problems in AM, will include the study of the composition distribution and stress, and the manufacture of objects with the optimal parameters obtained in this study. 

## Figures and Tables

**Table 1:** Summary of the conditions to produce a temperature increase of 300 °C.

Substrate Volume (cm^3^)	Heat (J)	Time (s)
5	3950	24.7

**Table 2:** Summary of the initial preheating parameters.

	Power (W)	Laser Spot (mm)	Scan Speed (mm/min)	Number of Scans
P1	1000	10	580	6

**Table 3:** Summary of the new conditions for the preheating.

	Power (W)	Laser Spot (mm)	Scan Speed (mm/min)	Number of Scans
P2	1000	10	580	4

**Table 4:** Summary of preheating parameters in simulation.

Power (W)	Laser Spot (mm)	Scan Speed (mm/min)	Number of Scans
400	10	580	4

**Table 5:** Summary of the simulation parameters for the melting step.

Power (W)	Laser Spot (mm)	Scan Speed (mm/min)
400	0.6	300

**Table 6:** Experimental conditions for preheating and melting.

	P_preh_ (W)	Spot_preh_ (mm)	Scan Speed_preh_ (mm/min)	Nº of Scans	P_melt_ (W)	Spot_melt_ (mm)	Scan Speed_melt_ (mm/min)
T1	1000	10	580	4	1000	0.6	300
T2	1000	10	580	4	500	0.6	150
T3	1000	10	580	4	300	0.6	90

**Table 7:** Chemical composition of as-received Al 7075 powder in wt. %.

Alloy	Al	Si	Fe	Cu	Mg	Zn	Cr	Ti	Mn
AA7075	Bal.	0.060	0.062	1.585	2.538	5.907	0.209	<0.005	<0.020

**Table 8:** Experimental tests for powder deposition of AA7075 alloy.

	P_preh_ (W)	Spot_preh_ (mm)	Scan Speed_preh_ (mm/min)	N° of Scans	P_melt_ (W)	Spot_melt_ (mm)	Scan Speed_melt_ (mm/min)	Powder Feed Rate_melt_ (g/min)
T4	1000	10	580	4	1000	0.6	300	1.4
T5	1000	10	580	4	1000	0.6	300	1.1
T6	1000	10	580	4	1000	0.6	300	0.8

**Table 9:** Maximum temperature in the thermocouples.

N° of Scans	Preheating Speed (mm/min)	Total Time (s)	T_cen_ (°C)	T_ext_ (°C)
6	580	24.8	514	448

**Table 10:** Average maximum temperatures for the experiments of four scans.

N° of Scans	Preheating Speed (mm/min)	Total Time (s)	T_cen_ (°C)	T_ext_ (°C)
4	580	16.55	409	357

**Table 11:** Width, height, and aspect ratio for each of the experimental conditions.

Powder Feed Rate (g/min)	Width (µm)	Height (µm)	Aspect Ratio
0.8	993	114	8.7
1.1	1015	149	6.8
1.4	1393	241	5.8

**Table 12:** Microhardness values of the deposited single tracks.

Zone	Microhardness (HV)
Melting pool	106 ± 2
HAZ	125 ± 3
Substrate	143 ± 5

## Data Availability

The data presented in this study are available on request from the corresponding author.
